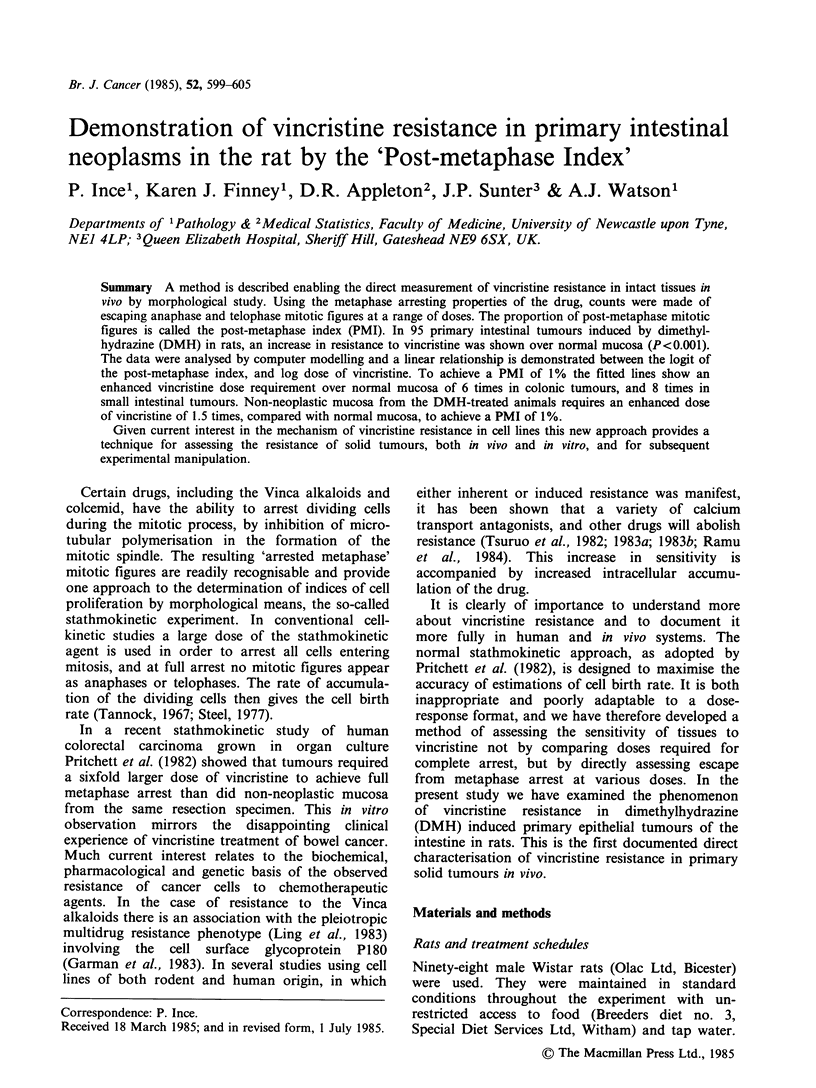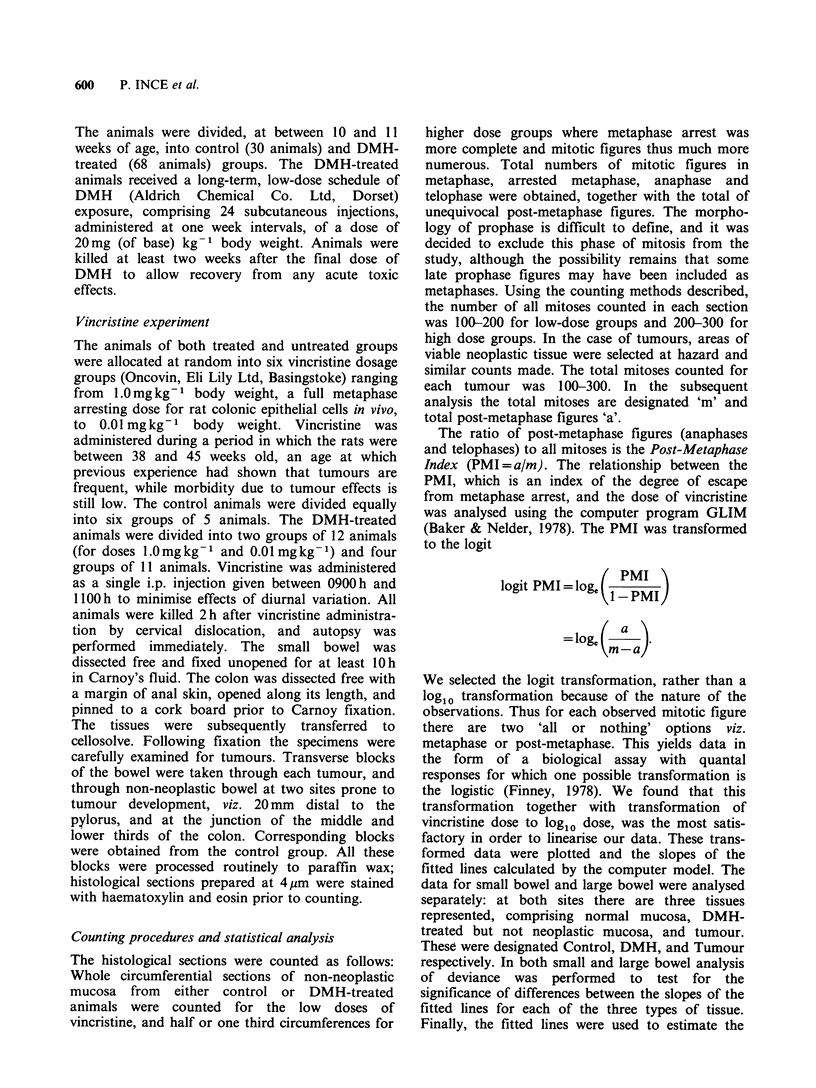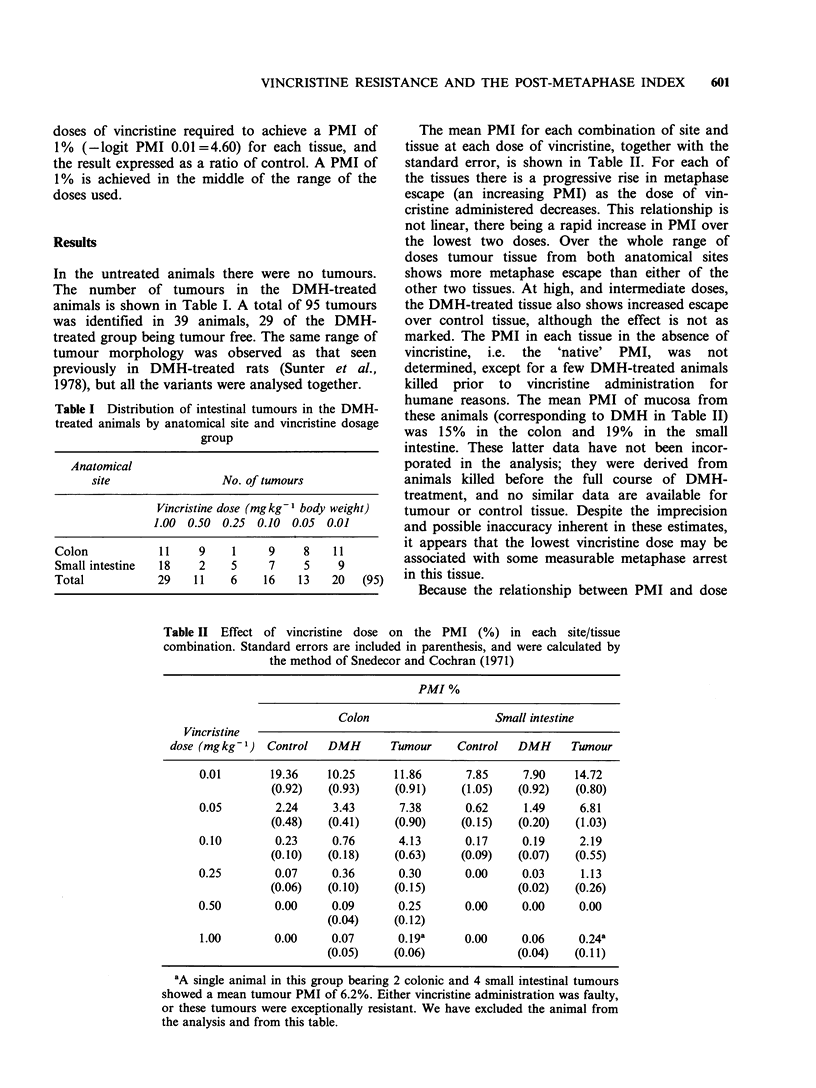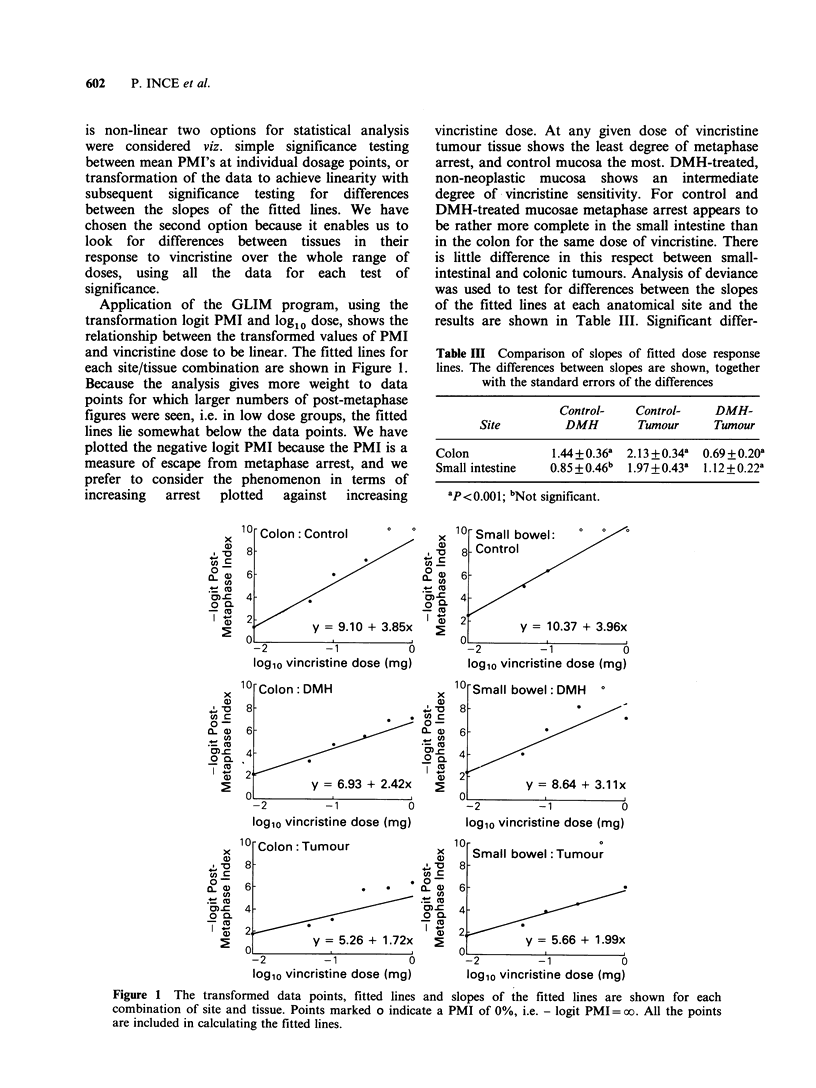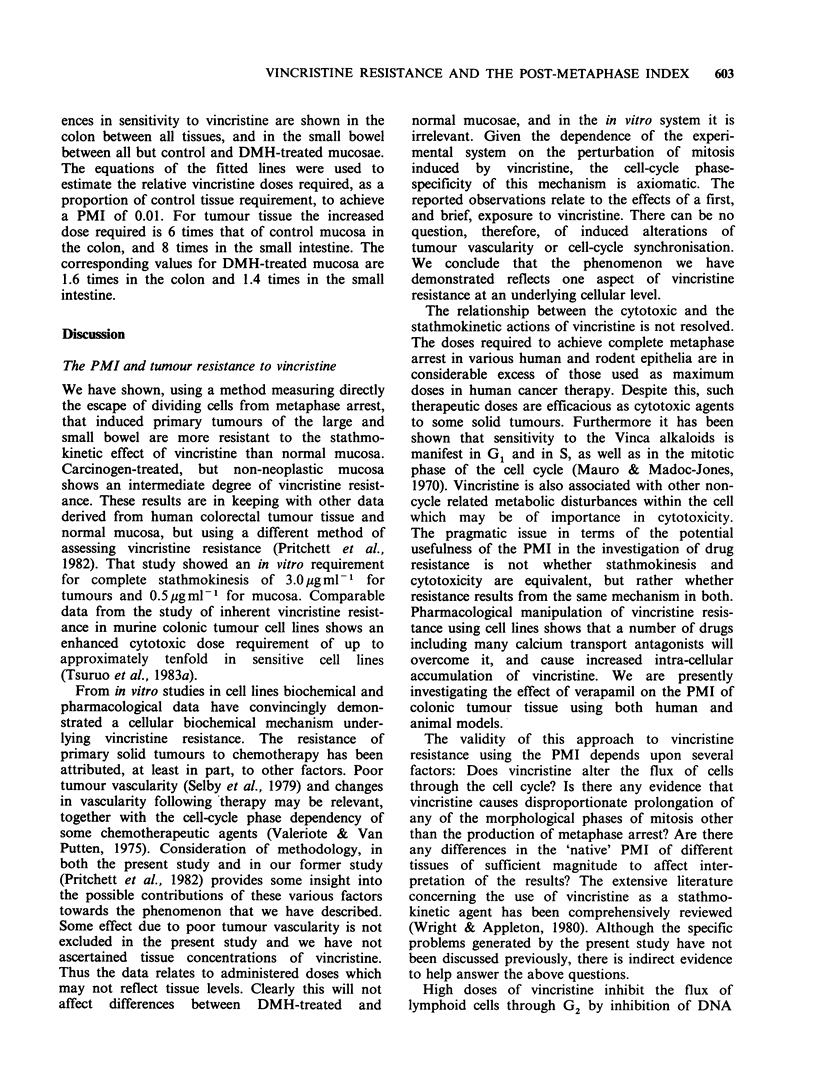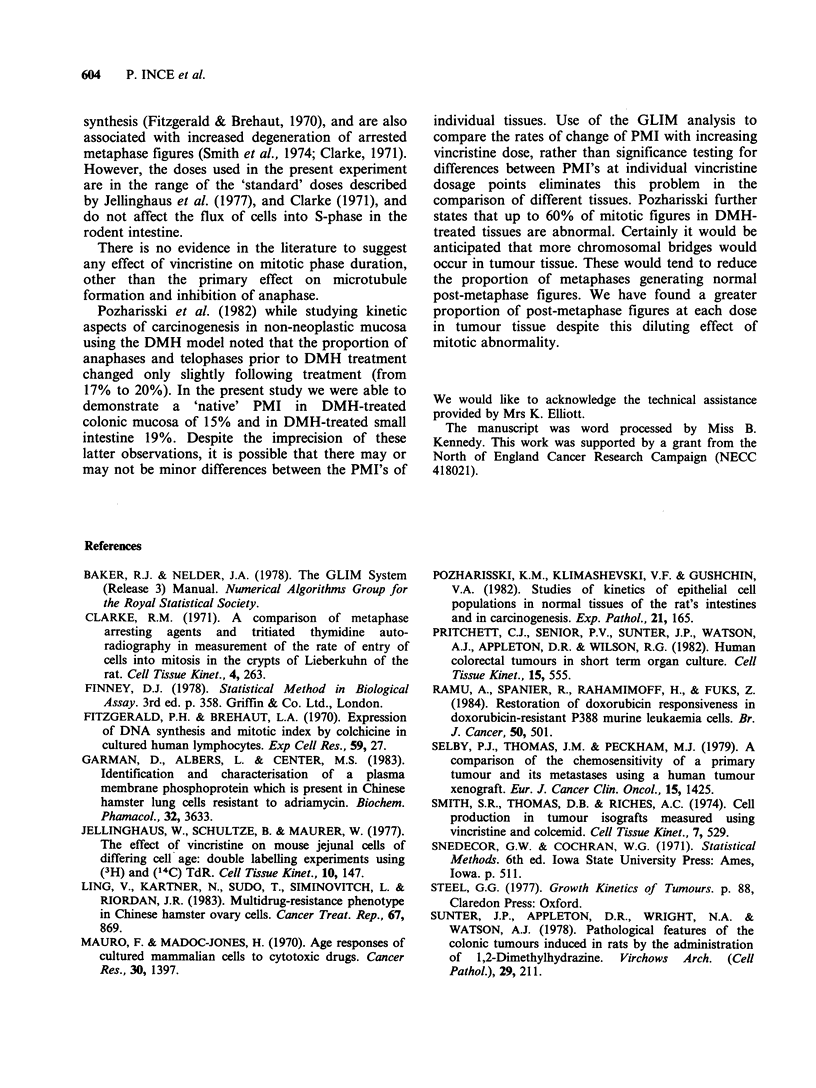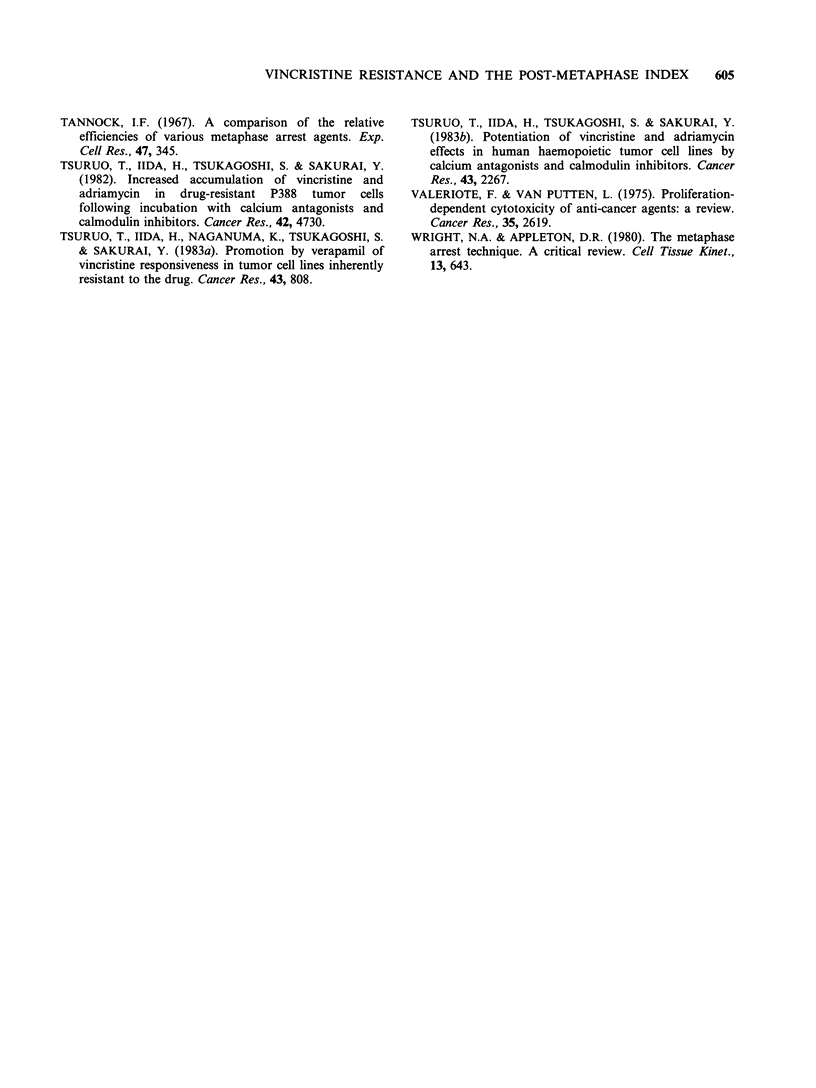# Demonstration of vincristine resistance in primary intestinal neoplasms in the rat by the 'post-metaphase index'.

**DOI:** 10.1038/bjc.1985.232

**Published:** 1985-10

**Authors:** P. Ince, K. J. Finney, D. R. Appleton, J. P. Sunter, A. J. Watson

## Abstract

A method is described enabling the direct measurement of vincristine resistance in intact tissues in vivo by morphological study. Using the metaphase arresting properties of the drug, counts were made of escaping anaphase and telophase mitotic figures at a range of doses. The proportion of post-metaphase mitotic figures is called the post-metaphase index (PMI). In 95 primary intestinal tumours induced by dimethylhydrazine (DMH) in rats, an increase in resistance to vincristine was shown over normal mucosa (P less than 0.001). The data were analysed by computer modelling and a linear relationship is demonstrated between the logit of the post-metaphase index, and log dose of vincristine. To achieve a PMI of 1% the fitted lines show an enhanced vincristine dose requirement over normal mucosa of 6 times in colonic tumours, and 8 times in small intestinal tumours. Non-neoplastic mucosa from the DMH-treated animals requires an enhanced dose of vincristine of 1.5 times, compared with normal mucosa, to achieve a PMI of 1%. Given current interest in the mechanism of vincristine resistance in cell lines this new approach provides a technique for assessing the resistance of solid tumours, both in vivo and in vitro, and for subsequent experimental manipulation.


Br. J. Cancer (1985), **52**, 599–605

### Demonstration of vincristine resistance in primary intestinal neoplasms in the rat by the 'Post-metaphase Index'

P. Ince[1], Karen J. Finney[1], D.R. Appleton[2], J.P. Sunter[3] & A.J. Watson[1]

*Departments of [1]Pathology & [2]Medical Statistics, Faculty of Medicine, University of Newcastle upon Tyne, NE1 4LP; [3]Queen Elizabeth Hospital, Sheriff Hill, Gateshead NE9 6SX, UK.*

**Summary** A method is described enabling the direct measurement of vincristine resistance in intact tissues *in vivo* by morphological study. Using the metaphase arresting properties of the drug, counts were made of escaping anaphase and telophase mitotic figures at a range of doses. The proportion of post-metaphase mitotic figures is called the post-metaphase index (PMI). In 95 primary intestinal tumours induced by dimethylhydrazine (DMH) in rats, an increase in resistance to vincristine was shown over normal mucosa ($P<0.001$). The data were analysed by computer modelling and a linear relationship is demonstrated between the logit of the post-metaphase index, and log dose of vincristine. To achieve a PMI of 1% the fitted lines show an enhanced vincristine dose requirement over normal mucosa of 6 times in colonic tumours, and 8 times in small intestinal tumours. Non-neoplastic mucosa from the DMH-treated animals requires an enhanced dose of vincristine of 1.5 times, compared with normal mucosa, to achieve a PMI of 1%.

Given current interest in the mechanism of vincristine resistance in cell lines this new approach provides a technique for assessing the resistance of solid tumours, both *in vivo* and *in vitro*, and for subsequent experimental manipulation.

Certain drugs, including the Vinca alkaloids and colcemid, have the ability to arrest dividing cells during the mitotic process, by inhibition of microtubular polymerisation in the formation of the mitotic spindle. The resulting 'arrested metaphase' mitotic figures are readily recognisable and provide one approach to the determination of indices of cell proliferation by morphological means, the so-called stathmokinetic experiment. In conventional cell-kinetic studies a large dose of the stathmokinetic agent is used in order to arrest all cells entering mitosis, and at full arrest no mitotic figures appear as anaphases or telophases. The rate of accumulation of the dividing cells then gives the cell birth rate (Tannock, 1967; Steel, 1977).

In a recent stathmokinetic study of human colorectal carcinoma grown in organ culture Pritchett *et al.* (1982) showed that tumours required a sixfold larger dose of vincristine to achieve full metaphase arrest than did non-neoplastic mucosa from the same resection specimen. This *in vitro* observation mirrors the disappointing clinical experience of vincristine treatment of bowel cancer. Much current interest relates to the biochemical, pharmacological and genetic basis of the observed resistance of cancer cells to chemotherapeutic agents. In the case of resistance to the Vinca alkaloids there is an association with the pleiotropic multidrug resistance phenotype (Ling *et al.*, 1983) involving the cell surface glycoprotein P180 (Garman *et al.*, 1983). In several studies using cell lines of both rodent and human origin, in which either inherent or induced resistance was manifest, it has been shown that a variety of calcium transport antagonists, and other drugs will abolish resistance (Tsuruo *et al.*, 1982; 1983*a*; 1983*b*; Ramu *et al.*, 1984). This increase in sensitivity is accompanied by increased intracellular accumulation of the drug.

It is clearly of importance to understand more about vincristine resistance and to document it more fully in human and *in vivo* systems. The normal stathmokinetic approach, as adopted by Pritchett *et al.* (1982), is designed to maximise the accuracy of estimations of cell birth rate. It is both inappropriate and poorly adaptable to a dose-response format, and we have therefore developed a method of assessing the sensitivity of tissues to vincristine not by comparing doses required for complete arrest, but by directly assessing escape from metaphase arrest at various doses. In the present study we have examined the phenomenon of vincristine resistance in dimethylhydrazine (DMH) induced primary epithelial tumours of the intestine in rats. This is the first documented direct characterisation of vincristine resistance in primary solid tumours *in vivo*.

### Materials and methods

### *Rats and treatment schedules*

Ninety-eight male Wistar rats (Olac Ltd, Bicester) were used. They were maintained in standard conditions throughout the experiment with unrestricted access to food (Breeders diet no. 3, Special Diet Services Ltd, Witham) and tap water.

Correspondence: P. Ince.
Received 18 March 1985; and in revised form, 1 July 1985.

© The Macmillan Press Ltd., 1985

The animals were divided, at between 10 and 11 weeks of age, into control (30 animals) and DMH-treated (68 animals) groups. The DMH-treated animals received a long-term, low-dose schedule of DMH (Aldrich Chemical Co. Ltd, Dorset) exposure, comprising 24 subcutaneous injections, administered at one week intervals, of a dose of 20 mg (of base) $kg^{-1}$ body weight. Animals were killed at least two weeks after the final dose of DMH to allow recovery from any acute toxic effects.

*Vincristine experiment*

The animals of both treated and untreated groups were allocated at random into six vincristine dosage groups (Oncovin, Eli Lily Ltd, Basingstoke) ranging from 1.0 mg $kg^{-1}$ body weight, a full metaphase arresting dose for rat colonic epithelial cells *in vivo*, to 0.01 mg $kg^{-1}$ body weight. Vincristine was administered during a period in which the rats were between 38 and 45 weeks old, an age at which previous experience had shown that tumours are frequent, while morbidity due to tumour effects is still low. The control animals were divided equally into six groups of 5 animals. The DMH-treated animals were divided into two groups of 12 animals (for doses 1.0 mg $kg^{-1}$ and 0.01 mg $kg^{-1}$) and four groups of 11 animals. Vincristine was administered as a single i.p. injection given between 0900 h and 1100 h to minimise effects of diurnal variation. All animals were killed 2 h after vincristine administration by cervical dislocation, and autopsy was performed immediately. The small bowel was dissected free and fixed unopened for at least 10 h in Carnoy's fluid. The colon was dissected free with a margin of anal skin, opened along its length, and pinned to a cork board prior to Carnoy fixation. The tissues were subsequently transferred to cellosolve. Following fixation the specimens were carefully examined for tumours. Transverse blocks of the bowel were taken through each tumour, and through non-neoplastic bowel at two sites prone to tumour development, *viz.* 20 mm distal to the pylorus, and at the junction of the middle and lower thirds of the colon. Corresponding blocks were obtained from the control group. All these blocks were processed routinely to paraffin wax; histological sections prepared at 4 $\mu$m were stained with haematoxylin and eosin prior to counting.

*Counting procedures and statistical analysis*

The histological sections were counted as follows: Whole circumferential sections of non-neoplastic mucosa from either control or DMH-treated animals were counted for the low doses of vincristine, and half or one third circumferences for higher dose groups where metaphase arrest was more complete and mitotic figures thus much more numerous. Total numbers of mitotic figures in metaphase, arrested metaphase, anaphase and telophase were obtained, together with the total of unequivocal post-metaphase figures. The morphology of prophase is difficult to define, and it was decided to exclude this phase of mitosis from the study, although the possibility remains that some late prophase figures may have been included as metaphases. Using the counting methods described, the number of all mitoses counted in each section was 100–200 for low-dose groups and 200–300 for high dose groups. In the case of tumours, areas of viable neoplastic tissue were selected at hazard and similar counts made. The total mitoses counted for each tumour was 100–300. In the subsequent analysis the total mitoses are designated 'm' and total post-metaphase figures 'a'.

The ratio of post-metaphase figures (anaphases and telophases) to all mitoses is the *Post-Metaphase Index* (PMI $= a/m$). The relationship between the PMI, which is an index of the degree of escape from metaphase arrest, and the dose of vincristine was analysed using the computer program GLIM (Baker & Nelder, 1978). The PMI was transformed to the logit

$$\text{logit PMI} = \log_e\left(\frac{\text{PMI}}{1-\text{PMI}}\right)$$

$$= \log_e\left(\frac{a}{m-a}\right).$$

We selected the logit transformation, rather than a $\log_{10}$ transformation because of the nature of the observations. Thus for each observed mitotic figure there are two 'all or nothing' options *viz.* metaphase or post-metaphase. This yields data in the form of a biological assay with quantal responses for which one possible transformation is the logistic (Finney, 1978). We found that this transformation together with transformation of vincristine dose to $\log_{10}$ dose, was the most satisfactory in order to linearise our data. These transformed data were plotted and the slopes of the fitted lines calculated by the computer model. The data for small bowel and large bowel were analysed separately: at both sites there are three tissues represented, comprising normal mucosa, DMH-treated but not neoplastic mucosa, and tumour. These were designated Control, DMH, and Tumour respectively. In both small and large bowel analysis of deviance was performed to test for the significance of differences between the slopes of the fitted lines for each of the three types of tissue. Finally, the fitted lines were used to estimate the doses of vincristine required to achieve a PMI of 1% (−logit PMI 0.01 = 4.60) for each tissue, and the result expressed as a ratio of control. A PMI of 1% is achieved in the middle of the range of the doses used.

### Results

In the untreated animals there were no tumours. The number of tumours in the DMH-treated animals is shown in Table I. A total of 95 tumours was identified in 39 animals, 29 of the DMH-treated group being tumour free. The same range of tumour morphology was observed as that seen previously in DMH-treated rats (Sunter *et al.*, 1978), but all the variants were analysed together.

**Table I** Distribution of intestinal tumours in the DMH-treated animals by anatomical site and vincristine dosage group

| *Anatomical site* | *No. of tumours* | | | | | | |
|---|---|---|---|---|---|---|---|
| | *Vincristine dose (mg kg$^{-1}$ body weight)* | | | | | | |
| | *1.00* | *0.50* | *0.25* | *0.10* | *0.05* | *0.01* | |
| Colon | 11 | 9 | 1 | 9 | 8 | 11 | |
| Small intestine | 18 | 2 | 5 | 7 | 5 | 9 | |
| Total | 29 | 11 | 6 | 16 | 13 | 20 | (95) |

The mean PMI for each combination of site and tissue at each dose of vincristine, together with the standard error, is shown in Table II. For each of the tissues there is a progressive rise in metaphase escape (an increasing PMI) as the dose of vincristine administered decreases. This relationship is not linear, there being a rapid increase in PMI over the lowest two doses. Over the whole range of doses tumour tissue from both anatomical sites shows more metaphase escape than either of the other two tissues. At high, and intermediate doses, the DMH-treated tissue also shows increased escape over control tissue, although the effect is not as marked. The PMI in each tissue in the absence of vincristine, i.e. the 'native' PMI, was not determined, except for a few DMH-treated animals killed prior to vincristine administration for humane reasons. The mean PMI of mucosa from these animals (corresponding to DMH in Table II) was 15% in the colon and 19% in the small intestine. These latter data have not been incorporated in the analysis; they were derived from animals killed before the full course of DMH-treatment, and no similar data are available for tumour or control tissue. Despite the imprecision and possible inaccuracy inherent in these estimates, it appears that the lowest vincristine dose may be associated with some measurable metaphase arrest in this tissue.

Because the relationship between PMI and dose

**Table II** Effect of vincristine dose on the PMI (%) in each site/tissue combination. Standard errors are included in parenthesis, and were calculated by the method of Snedecor and Cochran (1971)

| | *PMI %* | | | | | |
|---|---|---|---|---|---|---|
| | *Colon* | | | *Small intestine* | | |
| *Vincristine dose (mg kg$^{-1}$)* | *Control* | *DMH* | *Tumour* | *Control* | *DMH* | *Tumour* |
| 0.01 | 19.36 (0.92) | 10.25 (0.93) | 11.86 (0.91) | 7.85 (1.05) | 7.90 (0.92) | 14.72 (0.80) |
| 0.05 | 2.24 (0.48) | 3.43 (0.41) | 7.38 (0.90) | 0.62 (0.15) | 1.49 (0.20) | 6.81 (1.03) |
| 0.10 | 0.23 (0.10) | 0.76 (0.18) | 4.13 (0.63) | 0.17 (0.09) | 0.19 (0.07) | 2.19 (0.55) |
| 0.25 | 0.07 (0.06) | 0.36 (0.10) | 0.30 (0.15) | 0.00 | 0.03 (0.02) | 1.13 (0.26) |
| 0.50 | 0.00 | 0.09 (0.04) | 0.25 (0.12) | 0.00 | 0.00 | 0.00 |
| 1.00 | 0.00 | 0.07 (0.05) | 0.19[a] (0.06) | 0.00 | 0.06 (0.04) | 0.24[a] (0.11) |

[a]A single animal in this group bearing 2 colonic and 4 small intestinal tumours showed a mean tumour PMI of 6.2%. Either vincristine administration was faulty, or these tumours were exceptionally resistant. We have excluded the animal from the analysis and from this table.

is non-linear two options for statistical analysis were considered *viz.* simple significance testing between mean PMI's at individual dosage points, or transformation of the data to achieve linearity with subsequent significance testing for differences between the slopes of the fitted lines. We have chosen the second option because it enables us to look for differences between tissues in their response to vincristine over the whole range of doses, using all the data for each test of significance.

Application of the GLIM program, using the transformation logit PMI and $\log_{10}$ dose, shows the relationship between the transformed values of PMI and vincristine dose to be linear. The fitted lines for each site/tissue combination are shown in Figure 1. Because the analysis gives more weight to data points for which larger numbers of post-metaphase figures were seen, i.e. in low dose groups, the fitted lines lie somewhat below the data points. We have plotted the negative logit PMI because the PMI is a measure of escape from metaphase arrest, and we prefer to consider the phenomenon in terms of increasing arrest plotted against increasing vincristine dose. At any given dose of vincristine tumour tissue shows the least degree of metaphase arrest, and control mucosa the most. DMH-treated, non-neoplastic mucosa shows an intermediate degree of vincristine sensitivity. For control and DMH-treated mucosae metaphase arrest appears to be rather more complete in the small intestine than in the colon for the same dose of vincristine. There is little difference in this respect between small-intestinal and colonic tumours. Analysis of deviance was used to test for differences between the slopes of the fitted lines at each anatomical site and the results are shown in Table III. Significant differ-

**Table III** Comparison of slopes of fitted dose response lines. The differences between slopes are shown, together with the standard errors of the differences

| *Site* | *Control-DMH* | *Control-Tumour* | *DMH-Tumour* |
|---|---|---|---|
| Colon | $1.44 \pm 0.36$[a] | $2.13 \pm 0.34$[a] | $0.69 \pm 0.20$[a] |
| Small intestine | $0.85 \pm 0.46$[b] | $1.97 \pm 0.43$[a] | $1.12 \pm 0.22$[a] |

[a]$P < 0.001$; [b]Not significant.

**Figure 1** The transformed data points, fitted lines and slopes of the fitted lines are shown for each combination of site and tissue. Points marked o indicate a PMI of 0%, i.e. − logit PMI = ∞. All the points are included in calculating the fitted lines.

ences in sensitivity to vincristine are shown in the colon between all tissues, and in the small bowel between all but control and DMH-treated mucosae. The equations of the fitted lines were used to estimate the relative vincristine doses required, as a proportion of control tissue requirement, to achieve a PMI of 0.01. For tumour tissue the increased dose required is 6 times that of control mucosa in the colon, and 8 times in the small intestine. The corresponding values for DMH-treated mucosa are 1.6 times in the colon and 1.4 times in the small intestine.

### Discussion

### *The PMI and tumour resistance to vincristine*

We have shown, using a method measuring directly the escape of dividing cells from metaphase arrest, that induced primary tumours of the large and small bowel are more resistant to the stathmokinetic effect of vincristine than normal mucosa. Carcinogen-treated, but non-neoplastic mucosa shows an intermediate degree of vincristine resistance. These results are in keeping with other data derived from human colorectal tumour tissue and normal mucosa, but using a different method of assessing vincristine resistance (Pritchett *et al.*, 1982). That study showed an *in vitro* requirement for complete stathmokinesis of $3.0 \mu g ml^{-1}$ for tumours and $0.5 \mu g ml^{-1}$ for mucosa. Comparable data from the study of inherent vincristine resistance in murine colonic tumour cell lines shows an enhanced cytotoxic dose requirement of up to approximately tenfold in sensitive cell lines (Tsuruo *et al.*, 1983*a*).

From *in vitro* studies in cell lines biochemical and pharmacological data have convincingly demonstrated a cellular biochemical mechanism underlying vincristine resistance. The resistance of primary solid tumours to chemotherapy has been attributed, at least in part, to other factors. Poor tumour vascularity (Selby *et al.*, 1979) and changes in vascularity following therapy may be relevant, together with the cell-cycle phase dependency of some chemotherapeutic agents (Valeriote & Van Putten, 1975). Consideration of methodology, in both the present study and in our former study (Pritchett *et al.*, 1982) provides some insight into the possible contributions of these various factors towards the phenomenon that we have described. Some effect due to poor tumour vascularity is not excluded in the present study and we have not ascertained tissue concentrations of vincristine. Thus the data relates to administered doses which may not reflect tissue levels. Clearly this will not affect differences between DMH-treated and normal mucosae, and in the *in vitro* system it is irrelevant. Given the dependence of the experimental system on the perturbation of mitosis induced by vincristine, the cell-cycle phase-specificity of this mechanism is axiomatic. The reported observations relate to the effects of a first, and brief, exposure to vincristine. There can be no question, therefore, of induced alterations of tumour vascularity or cell-cycle synchronisation. We conclude that the phenomenon we have demonstrated reflects one aspect of vincristine resistance at an underlying cellular level.

The relationship between the cytotoxic and the stathmokinetic actions of vincristine is not resolved. The doses required to achieve complete metaphase arrest in various human and rodent epithelia are in considerable excess of those used as maximum doses in human cancer therapy. Despite this, such therapeutic doses are efficacious as cytotoxic agents to some solid tumours. Furthermore it has been shown that sensitivity to the Vinca alkaloids is manifest in $G_1$ and in S, as well as in the mitotic phase of the cell cycle (Mauro & Madoc-Jones, 1970). Vincristine is also associated with other non-cycle related metabolic disturbances within the cell which may be of importance in cytotoxicity. The pragmatic issue in terms of the potential usefulness of the PMI in the investigation of drug resistance is not whether stathmokinesis and cytotoxicity are equivalent, but rather whether resistance results from the same mechanism in both. Pharmacological manipulation of vincristine resistance using cell lines shows that a number of drugs including many calcium transport antagonists will overcome it, and cause increased intra-cellular accumulation of vincristine. We are presently investigating the effect of verapamil on the PMI of colonic tumour tissue using both human and animal models.

The validity of this approach to vincristine resistance using the PMI depends upon several factors: Does vincristine alter the flux of cells through the cell cycle? Is there any evidence that vincristine causes disproportionate prolongation of any of the morphological phases of mitosis other than the production of metaphase arrest? Are there any differences in the 'native' PMI of different tissues of sufficient magnitude to affect interpretation of the results? The extensive literature concerning the use of vincristine as a stathmokinetic agent has been comprehensively reviewed (Wright & Appleton, 1980). Although the specific problems generated by the present study have not been discussed previously, there is indirect evidence to help answer the above questions.

High doses of vincristine inhibit the flux of lymphoid cells through $G_2$ by inhibition of DNA

synthesis (Fitzgerald & Brehaut, 1970), and are also associated with increased degeneration of arrested metaphase figures (Smith *et al.*, 1974; Clarke, 1971). However, the doses used in the present experiment are in the range of the 'standard' doses described by Jellinghaus *et al.* (1977), and Clarke (1971), and do not affect the flux of cells into S-phase in the rodent intestine.

There is no evidence in the literature to suggest any effect of vincristine on mitotic phase duration, other than the primary effect on microtubule formation and inhibition of anaphase.

Pozharisski *et al.* (1982) while studying kinetic aspects of carcinogenesis in non-neoplastic mucosa using the DMH model noted that the proportion of anaphases and telophases prior to DMH treatment changed only slightly following treatment (from 17% to 20%). In the present study we were able to demonstrate a 'native' PMI in DMH-treated colonic mucosa of 15% and in DMH-treated small intestine 19%. Despite the imprecision of these latter observations, it is possible that there may or may not be minor differences between the PMI's of individual tissues. Use of the GLIM analysis to compare the rates of change of PMI with increasing vincristine dose, rather than significance testing for differences between PMI's at individual vincristine dosage points eliminates this problem in the comparison of different tissues. Pozharisski further states that up to 60% of mitotic figures in DMH-treated tissues are abnormal. Certainly it would be anticipated that more chromosomal bridges would occur in tumour tissue. These would tend to reduce the proportion of metaphases generating normal post-metaphase figures. We have found a greater proportion of post-metaphase figures at each dose in tumour tissue despite this diluting effect of mitotic abnormality.

We would like to acknowledge the technical assistance provided by Mrs K. Elliott.

The manuscript was word processed by Miss B. Kennedy. This work was supported by a grant from the North of England Cancer Research Campaign (NECC 418021).

**References**

BAKER, R.J. & NELDER, J.A. (1978). The GLIM System (Release 3) Manual. *Numerical Algorithms Group for the Royal Statistical Society.*

CLARKE, R.M. (1971). A comparison of metaphase arresting agents and tritiated thymidine autoradiography in measurement of the rate of entry of cells into mitosis in the crypts of Lieberkuhn of the rat. *Cell Tissue Kinet.*, **4**, 263.

FINNEY, D.J. (1978). *Statistical Method in Biological Assay.* 3rd ed. p. 358. Griffin & Co. Ltd., London.

FITZGERALD, P.H. & BREHAUT, L.A. (1970). Expression of DNA synthesis and mitotic index by colchicine in cultured human lymphocytes. *Exp Cell Res.*, **59**, 27.

GARMAN, D., ALBERS, L. & CENTER, M.S. (1983). Identification and characterisation of a plasma membrane phosphoprotein which is present in Chinese hamster lung cells resistant to adriamycin. *Biochem. Phamacol.*, **32**, 3633.

JELLINGHAUS, W., SCHULTZE, B. & MAURER, W. (1977). The effect of vincristine on mouse jejunal cells of differing cell age: double labelling experiments using ($^{3}H$) and ($^{14}C$) TdR. *Cell Tissue Kinet.*, **10**, 147.

LING, V., KARTNER, N., SUDO, T., SIMINOVITCH, L. & RIORDAN, J.R. (1983). Multidrug-resistance phenotype in Chinese hamster ovary cells. *Cancer Treat. Rep.*, **67**, 869.

MAURO, F. & MADOC-JONES, H. (1970). Age responses of cultured mammalian cells to cytotoxic drugs. *Cancer Res.*, **30**, 1397.

POZHARISSKI, K.M., KLIMASHEVSKI, V.F. & GUSHCHIN, V.A. (1982). Studies of kinetics of epithelial cell populations in normal tissues of the rat's intestines and in carcinogenesis. *Exp. Pathol.*, **21**, 165.

PRITCHETT, C.J., SENIOR, P.V., SUNTER, J.P., WATSON, A.J., APPLETON, D.R. & WILSON, R.G. (1982). Human colorectal tumours in short term organ culture. *Cell Tissue Kinet.*, **15**, 555.

RAMU, A., SPANIER, R., RAHAMIMOFF, H., & FUKS, Z. (1984). Restoration of doxorubicin responsiveness in doxorubicin-resistant P388 murine leukaemia cells. *Br. J. Cancer*, **50**, 501.

SELBY, P.J., THOMAS, J.M. & PECKHAM, M.J. (1979). A comparison of the chemosensitivity of a primary tumour and its metastases using a human tumour xenograft. *Eur. J. Cancer Clin. Oncol.*, **15**, 1425.

SMITH, S.R., THOMAS, D.B. & RICHES, A.C. (1974). Cell production in tumour isografts measured using vincristine and colcemid. *Cell Tissue Kinet.*, **7**, 529.

SNEDECOR, G.W. & COCHRAN, W.G. (1971). *Statistical Methods.* 6th ed. Iowa State University Press: Ames, Iowa. p. 511.

STEEL, G.G. (1977). *Growth Kinetics of Tumours.* p. 88, Claredon Press: Oxford.

SUNTER, J.P., APPLETON, D.R., WRIGHT, N.A. & WATSON, A.J. (1978). Pathological features of the colonic tumours induced in rats by the administration of 1,2-Dimethylhydrazine. *Virchows Arch.* (*Cell Pathol.*), **29**, 211.

TANNOCK, I.F. (1967). A comparison of the relative efficiencies of various metaphase arrest agents. *Exp. Cell Res.*, **47**, 345.

TSURUO, T., IIDA, H., TSUKAGOSHI, S. & SAKURAI, Y. (1982). Increased accumulation of vincristine and adriamycin in drug-resistant P388 tumor cells following incubation with calcium antagonists and calmodulin inhibitors. *Cancer Res.*, **42**, 4730.

TSURUO, T., IIDA, H., NAGANUMA, K., TSUKAGOSHI, S. & SAKURAI, Y. (1983*a*). Promotion by verapamil of vincristine responsiveness in tumor cell lines inherently resistant to the drug. *Cancer Res.*, **43**, 808.

TSURUO, T., IIDA, H., TSUKAGOSHI, S. & SAKURAI, Y. (1983*b*). Potentiation of vincristine and adriamycin effects in human haemopoietic tumor cell lines by calcium antagonists and calmodulin inhibitors. *Cancer Res.*, **43**, 2267.

VALERIOTE, F. & VAN PUTTEN, L. (1975). Proliferation-dependent cytotoxicity of anti-cancer agents: a review. *Cancer Res.*, **35**, 2619.

WRIGHT, N.A. & APPLETON, D.R. (1980). The metaphase arrest technique. A critical review. *Cell Tissue Kinet.*, **13**, 643.